# The global burden of HIV-1 drug resistance in the past 20 years

**DOI:** 10.7717/peerj.4848

**Published:** 2018-05-25

**Authors:** Maurizio Zazzi, Hui Hu, Mattia Prosperi

**Affiliations:** 1 Department of Medical Biotechnologies, University of Siena, Italy; 2 Department of Epidemiology, University of Florida, United States of America

**Keywords:** Antiretroviral treatment, Genotypic interpretation system, HIV/AIDS, HIV subtype, Drug resistance

## Abstract

Genotypic drug resistance testing has been an integral part of the clinical management of HIV patients for almost 20 years, not only assisting treatment choices but also informing drug development. Accurate estimations on the worldwide circulation of drug resistance are difficult to obtain, particularly in low/middle-income countries. In this work, we queried two of the largest public HIV sequence repositories in the world—Los Alamos and Stanford HIVdb—to derive global prevalence, time trends and geodemographic predictors of HIV drug resistance. Different genotypic interpretation systems were used to ascertain resistance to reverse transcriptase and protease inhibitors. Continental, subtype-specific (including circulating recombinant forms) stratification as well as analysis on drug-naïve isolates were performed. Geographic information system analysis correlated country-specific drug resistance to sociodemographic and health indicators obtained from the World Bank. By looking at over 33,000 sequences worldwide between 1996 and 2016, increasing drug resistance trends with non-B subtypes and recombinants were found; transmitted drug resistance appeared to remain stable in the last decade. While an increase in drug resistance is expected with antiretroviral therapy rollout in resource-constrained areas, the plateau effect in areas covered by the most modern drug regimens warns against the downgrading of the resistance issue.

## Introduction

Development of drug resistance has been hampering antiretroviral therapy (ART) since the availability of the first anti-HIV drug. Indeed, genotypic drug resistance testing has been an integral part of the clinical management of HIV patients for almost 20 years, not only assisting treatment choices for individual patients but also informing drug development to deliver new drugs and novel drug classes to combat drug-resistant virus selected by previous treatments (Clutter et al., 2016; Iyidogan & Anderson, 2014). HIV drug resistance genotyping is probably the best example of the impact of molecular diagnostics on treatment of an infectious disease. As expected, progress in ART has been paralleled by changes in the prevalence and circulation of different drug-resistant variants along with availability and use of new drugs, drug classes and drug combinations. Notably and reassuringly, most currently recommended drug regimens bring together potency, tolerability, ease of adherence and particularly a medium-to-high genetic barrier to resistance. As a result, the prevalence and incidence of emergent drug resistance have declined in recent years in high-income countries where the most modern treatment regimens have been increasingly used (Frentz et al., 2014; Rhee et al., 2015; Schultze et al., 2015). On the other hand, the ART rollout in low/middle-income countries has progressively expanded the number of patients under therapy but less tolerable and lower genetic barrier regimens have been used widely, actually creating the prerequisites for selection of drug-resistant variants (Bertagnolio et al., 2012). Indeed, the prevalence of drug resistance has been reported to increase significantly in several low/middle-countries in the last few years (Pham et al., 2014).

The relevance of HIV drug resistance would advise for some kind of regularly updated and structured worldwide surveillance to detect new trends and cope with issues timely. However, accurate information on the circulation of drug resistance is difficult to obtain and update, particularly, but not exclusively, in low/middle-income countries and global figures can only be estimated from temporally and geographically sparse data (Godfrey et al., 2017; Minior et al., 2017; Singh et al., 2014). In this work, we queried the Los Alamos HIV data base to derive the worldwide prevalence, time trends and geodemographic predictors of HIV drug resistance, and used the Stanford HIV drug resistance repository for sensitivity analysis. With the aim of estimating the global burden of HIV drug resistance, the primary analysis was done on cumulative resistance, i.e., emergent (on treatment or acquired) and transmitted (pretreatment or primary) resistance. However, given its importance per se, transmitted resistance was also separately investigated.

## Methods

This is a secondary data analysis on public data, collating individual-level de-identified demographic information associated to HIV gene sequences. The authors assert that all procedures contributing to this work comply with the ethical standards of the relevant national and institutional committees on human experimentation and with the Helsinki Declaration of 1975, as revised in 2008.

We set up an advanced query on the Los Alamos HIV data base (Los Alamos National Security LLC, 2017), selecting HIV-1 sequences of any subtype with a known sample year between 1996 and 2016, encompassing the whole protease region (1–99 amino acids) and at least the first 250 amino acids of the reverse transcriptase, using HXB2 nucleotide numbering as reference. Problematic sequences—which as per the Los Alamos definition include high content of ambiguous bases, G–A hypermutants, synthetic or contaminants—were excluded. As additional background information, we selected the Genbank accession number, patient identifier, treatment status, age, sex, mode of HIV transmission, geographic region (continental area as per the Los Alamos definition), and country. Patients with unknown or non-uniquely ascertainable patient identifier were excluded, and only one sequence per patient per year was retained, with no restrictions on the infection time, choosing the earliest sequence in case of multiple entries available in a year.

All downloaded sequences were submitted to Stanford’s HIVdb web service (Stanford University, 2017) for quality check, alignment/extraction of mutations with respect to subtype B consensus, and calculation of drug resistance to nucleotide/nucleoside reverse transcriptase inhibitors (NRTIs), non-nucleoside reverse transcriptase inhibitors (NNRTIs), and protease inhibitors (PIs) using the three available algorithms of ANRS v.26 (France) (HIV French Resistance, 2017), Rega v.9.1 (Belgium) (Ku Leuven, 2017), and HIVdb v.8.4 (USA) (Stanford University, 2017). Resistance to one single drug was defined as an intermediate/resistant scoring as per the Stanford’s standardized scale, including non-ritonavir boosted PI scoring. Resistance to a drug class (NRTI/NNRTI/PI) was defined as standardized intermediate/resistant scoring for at least one drug belonging to that class. Per-drug and per-drug-class rate of agreement among the three scoring systems was calculated via Cohen’s kappa. Transmitted drug resistance was estimated separately using the WHO 2009 list of surveillance drug-resistance mutations (Gifford et al., 2009). Given the large number of sequences involved, fast subtyping was performed with BLAST on the latest HIV subtype and recombinant form reference set available on the Los Alamos data base, composed of 60 subtypes of which 14 pure and 46 circulating recombinant forms (CRFs), plus the chimpanzee simian immunodeficiency virus outgroup; two-three representatives per subtype are available, for a total of 170 reference sequences. For comparison, a subset of sequences was also subtyped using the Rega subtyping tool v.3 (Pineda-Pena et al., 2013).

We analyzed spatiotemporal trends in prevalence of drug resistance to NRTI/NNRTI and PI classes in relation to subtype, geographic area, and sociodemographic characteristics. We used descriptive, univariate and multivariate (logistic regression) analysis, and geographic information system (GIS) analysis. For GIS analysis, prevalence of B subtype, NNRTI, NRTI, PI, and two-class resistance were calculated for countries with at least 50 sequences from 2006 to 2016. Maps were generated using the ArcGIS 10.3 (ESRI, 2017). Country-level sociodemographic and health indicators were obtained from the World Bank’s DataBank (The World Bank, 2017). We retrieved the average values of the following 22 indicators between 2006 and 2016: (1) Adjusted net national income per capita (constant 2010 USD), (2) Health expenditure, total (% of GDP), (3) Health expenditure, public (% of GDP), (4) Adequacy of social insurance programs (% of total welfare of beneficiary households), (5) Adults (ages 15+) and children (ages 0–14) newly infected with HIV, (6) Antiretroviral therapy coverage (% of people living with HIV), (7) Condom use, population ages 15–24, female (% of females ages 15–24), (8) Condom use, population ages 15–24, male (% of males ages 15–24), (9) CPIA gender equality rating (1 = low to 6 = high), (10) Improved sanitation facilities (% of population with access), (11) Incidence of tuberculosis (per 100,000 people), (12) Literacy rate, adult total (% of people ages 15 and above), (13) Physicians (per 1000 people), (14) Population ages 15–64 (% of total), (15) Population growth (annual %), (16) Poverty gap at national poverty lines (%), (17) Prevalence of HIV, total (% of population ages 15–49), (18) Prevalence of undernourishment (% of population), (19) Strength of legal rights index (0 = weak to 12 = strong), (20) Teenage mothers (% of women ages 15–19 who have had children or are currently pregnant), (21) Total alcohol consumption per capita (liters of pure alcohol, projected estimates, 15+ years of age), and (22) Unemployment, total (% of total labor force, modeled ILO estimate). Spearman correlations were calculated between these indicators and the prevalence of B subtype, NNRTI, NRTI, PI, and the two-class resistance. Heatmaps were generated using the ggplot2 package in R (R Development Core Team, 2008).

In a sensitivity analysis, we downloaded HIVdb’s Genotype-rx datasets, which include all publicly available HIV-1 isolates in HIVdb and the lists of antiretrovirals received prior to the isolations. These data sets are well curated in terms of sequence quality control and treatment status, but lack patient-level demographic information (e.g., gender), with only year and country available. Therefore we used them to confirm drug resistance trends across classes over time.

## Results

We downloaded 107,820 sequences meeting the inclusion criteria from the Los Alamos data base, of which 99.5% passed the HIVdb/BLAST quality check. Non-ambiguous patient identifier and known geographic area was retrieved for 33,057 instances. Table 1 shows population characteristics for the filtered data stratified by geographic region. Half of the sequence isolates came from China, United States, South Africa, Germany and United Kingdom. There was uneven distribution of sequences per country across calendar years (more sequences from North America in earliest years). About 37% of sequences were from antiretroviral-naïve people, evenly distributed across continental areas with the sole exception of North America (6%). The prevalence of B subtypes was 38%, with subtype C being the most prevalent among non-B/CRF ones (23%). Concordance between BLAST and Rega v.3 was high (94% on a subsample of 1,000 sequences), with major discrepancies found on complex recombinant forms (also due to the different reference data sets used).

The worldwide resistance prevalence between 1996 and 2016 was about 12% for PIs, 21% for NRTIs and 22% for NNRTIs, using Stanford’s HIVdb algorithm.

The rate of agreement among the three genotypic resistance interpretation algorithms was high overall for each single drug, with the exception of non-B subtypes and PIs, for which ANRS has a completely different score distribution as compared to HIVdb and Rega; the problem is known and relates to tipranavir interpretation. Overall, all algorithms showed high rates of agreement, between 90% and 97%, the highest rates being between HIVdb and Rega (Table S1). Some relevant lower agreement included ANRS and HIVdb/Rega in regards to NNRTIs with subtype B (81%–83%), Rega and HIVdb for PIs with non-B subtypes/CRFs (75%), and as expected ANRS and HIVdb/Rega for PIs with non-B subtypes/CRFs (1%).

**Table 1:** Characteristics of the HIV-1 isolates retrieved from the Los Alamos sequence data base. Data are stratified by continental area (one sequence per person per year, encompassing 1–99 amino acids of the protease and 1–250 of the reverse transcriptase genes, all with a known year and geographic origin).

Data attribute/ Continental area	World	Africa	Asia/Oceania	Central/South America and Caribbean	Europe/Middle East/Former USSR and Russian Federation	North America
N (%)	33057 (100%)	9100 (27.5%)	9222 (27.9%)	1471 (4.4%)	9135 (27.6%)	4129 (12.5%)
Top-5 countries	China 5789 (17.5%) United States 4087 (12.4%) South Africa 3187 (9.6%) Germany 2179 (6.6%) United Kingdom 1189 (3.6%)	South Africa 3187 (35.0%) Uganda 1103 (12.1%) Zambia 860 (9.5%) Botswana 706 (7.8%) Cameroon 493 (5.4%)	China 5789 (62.8%) India 803 (8.7%) Japan 447 (4.8%) Australia 427 (4.6%) Thailand 372 (4%)	Brazil 591 (40.2%) Venezuela 206 (14%) Honduras 182 (12.4%) Argentina 158 (10.7%) Cuba 142 (9.7%)	Germany 2179 (23.9%) United Kingdom 1189 (13%) Russian Federation 1115 (12.2%) Spain 1112 (12.2%) Poland 960 (10.5%)	United States 4087 (99.0%) Canada 42 (1.0%)
Top-5 subtypes	B 12508 (37.8%) C 7700 (23.3%) 01_AE 3911 (11.8%) 07_BC 1720 (5.2%) 02_AG 1225 (3.7%)	C 5938 (65.3%) A1 756 (8.3%) D 643 (7.1%) 02_AG 561 (6.2%) 01_AE 189 (2.1%)	01_AE 3031 (32.9%) B 1996 (21.6%) 07_BC 1713 (18.6%) C 1106 (12%) 15_01B 533 (5.8%)	B 972 (66.1%) C 110 (7.5%) 12_BF 83 (5.6%) F1 76 (5.2%) 19_cpx 26 (1.8%)	B 6120 (67%) 01_AE 602 (6.6%) 15_01B 459 (5%) 02_AG 452 (4.9%) F1 286 (3.1%)	B 3298 (79.9%) C 268 (6.5%) 02_AG 157 (3.8%) A1 110 (2.7%) 01_AE 89 (2.2%)
Sex						
F	6880 (20.8%)	4017 (44.1%)	1153 (12.5%)	306 (20.8%)	828 (9.1%)	576 (13.9%)
M	13452 (40.7%)	1781 (19.6%)	5504 (59.7%)	381 (25.9%)	4614 (50.5%)	1172 (28.4%)
Unknown	12725 (38.5%)	3302 (36.3%)	2565 (27.8%)	784 (53.3%)	3693 (40.4%)	2381 (57.7%)
Age years						
26 to 33	1316 (4.0%)	522 (5.7%)	318 (3.4%)	54 (3.7%)	223 (2.4%)	199 (4.8%)
34 to 44	3294 (10.0%)	1586 (17.4%)	697 (7.6%)	139 (9.4%)	467 (5.1%)	405 (9.8%)
Above 44	1000 (3.0%)	333 (3.7%)	135 (1.5%)	29 (2%)	184 (2%)	319 (7.7%)
Below 26	1839 (5.6%)	1090 (12%)	317 (3.4%)	115 (7.8%)	130 (1.4%)	187 (4.5%)
Unknown	25608 (77.5%)	5569 (61.2%)	7755 (84.1%)	1134 (77.1%)	8131 (89%)	3019 (73.1%)
Mode of HIV transmission						
Heterosexual	4096 (12.4%)	1328 (14.6%)	1454 (15.8%)	300 (20.4%)	948 (10.4%)	66 (1.6%)
Homosexual	7619 (23.0%)	214 (2.4%)	3296 (35.7%)	226 (15.4%)	3297 (36.1%)	586 (14.2%)
Intravenous drug user	2142 (6.5%)	0 (0%)	1492 (16.2%)	15 (1%)	597 (6.5%)	38 (0.9%)
Mother-to-child	1384 (4.2%)	1051 (11.5%)	37 (0.4%)	173 (11.8%)	99 (1.1%)	24 (0.6%)
Other/Unknown	17496 (52.9%)	6456 (70.9%)	2754 (29.9%)	686 (46.6%)	4185 (45.8%)	3415 (82.7%)
Sex worker	320 (1.0%)	51 (0.6%)	189 (2%)	71 (4.8%)	9 (0.1%)	0 (0%)
Antiretroviral treatment-naïve	12164 (36.8%)	3353 (36.8%)	3797 (41.2%)	514 (34.9%)	4249 (46.5%)	251 (6.1%)
Drug resistance						
NRTI (all years)	6934 (21%)	2829 (31.0%)	768 (8.3%)	292 (19.9%)	2318 (25.4%)	1848 (44.8%)
NNRTI (all years)	7244 (21.9%)	430 (4.7%)	277 (3%)	310 (21.1%)	2111 (23.1%)	1226 (29.7%)
PI (all years)	3887 (11.8%)	5721 (17.3%)	1788 (19.6%)	173 (11.8%)	1772 (19.4%)	1235 (29.9%)
Two or more classes (all years)	2829 (14.3%)	1749 (32.9%)	321 (3.5%)	235 (16%)	1911 (20.9%)	1466 (35.5%)
NRTI (2007–2016)	3836 (19.3%)	2227 (41.9%)	329 (4.2%)	147 (18.4%)	385 (8.5%)	219 (15.7%)
NNRTI (2007–2016)	874 (4.4%)	310 (5.8%)	576 (7.4%)	156 (19.5%)	552 (12.2%)	325 (23.4%)
PI (2007–2016)	2300 (11.6%)	1599 (30.1%)	203 (2.6%)	92 (11.5%)	169 (3.7%)	100 (7.2%)
Two or more classes (2007–2016)	1998 (22.0%)	478 (5.2%)	220 (2.8%)	110 (13.8%)	211 (4.7%)	160 (11.5%)
Calendar Years						
Top-3 Years	2007/2008/2009 (28.7%)	2006/2007/2009 (33.4%)	2007/2009/2012 (40.7%)	2004/2008/2009 (40.1%)	2003/2006/2012 (16.7%)	1998/1999/2000 (52.9%)
Median (Interquartile Range)	2008 (2004–2010)	2007 (2005–2010)	2009 (2007–2012)	2007 (2004–2009)	2006 (2003–2011)	2000 (1999–2008)

From now on, we will use the HIVdb interpretation as proxy for drug resistance among NRTI/NNRTI/PI classes, given the high agreement with Rega, which means that for all drugs at least two algorithms were concordant in resistance interpretation.

**Figure 1:**
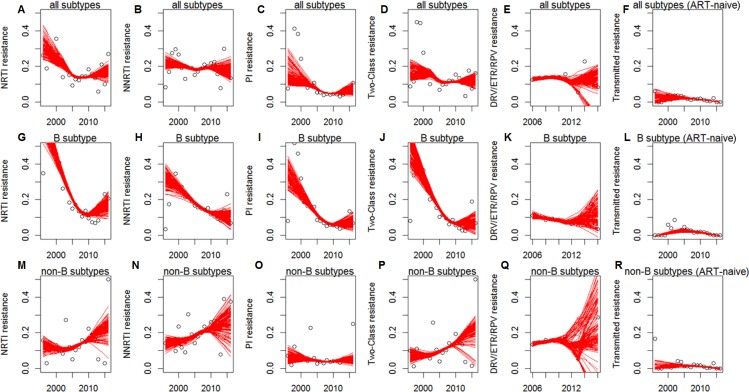
Prevalence of NRTI, NNRTI, PI, two-class, DRV/ETR/RPV, and transmitted drug resistance in therapy-naïve people, by calendar year in all subtypes (A–F) and in B (G–L) vs. non-B subtypes or circulating recombinant forms (M–R). Point estimates indicate per-year prevalence, whilst line estimates are drawn by lowess interpolation and data bootstrapping (150 times).

Figure 1 plots the worldwide trends (1996 to 2016) in HIV drug resistance to NRTIs/NNRTIs/PIs, multi-class and newest NNRTI/PI drugs, overall and stratified per B vs. non-B subtypes/CRFs. There was a sharp decrease in resistance prevalence across all drug classes in subtype B sequences, flattening in the last decade. Instead, resistance to NRTIs, NNRTIs and two-class resistance increased in non-B subtypes/CRFs (remaining stable with respect to PIs). Transmitted drug resistance seemed to remain low and stable (below 8%) in all subtypes across years. Resistance to newest NNRTIs and PIs (etravirine, rilpivirine, darunavir) seem to increase over the years in non-B subtypes/CRFs, although more recent years exhibit higher uncertainty.

Notably, the worldwide resistance trends were consistent with the estimation made on HIVdb’s Genotype-rx datasets (Figure S1). After filtering, the Genotype-rx data sets had a larger sample size (64746 isolates spanning reverse transcriptase and protease). Compared to the Los Alamos dataset, there was a higher proportion of subtype B isolates (51%), and different continental/national coverages (with the top-frequency country being Brazil).

When looking at geodemographic factors associated to drug resistance in the Los Alamos dataset (Table 2), we found that drug resistance to NRTIs and PIs has been decreasing since 1996 (OR 0.95, 95% CI [0.94–0.96], *p*-value < 0.0001 for NRTIs; and 0.92, 95% CI [0.91–0.92], *p*-value < 0.0001 for PIs), whilst an apparent increase in NNRTIs resistance was detected (OR 1.03 per 10-years increase, 95% CI [1.02–1.04], *p*-value < 0.0001).

**Table 2:** Geodemographic factors associated with genotypic resistance to HIV-1 NRTIs, NNRTIs, PIs, and to multi-class resistance.

Data attribute/Resistance to drug class	NRTI	NNRTI	PI	Two or more classes	Any among DRV, ETR, RPV (2006–2016)
	Odds ratio (95% Confidence Interval) [*P*-value]				
Sex M vs. F	1 (0.99–1.02) [0.729]	0.97 (0.96–0.98) [<0.0001]	0.98 (0.97–0.99) [0.0008]	0.99 (0.98–1.01) [0.2976]	0.99 (0.98–1.01) [0.2966]
Sex unknown vs. F	1.1 (1.09–1.12) [<0.0001]	1.03 (1.02–1.04) [<0.0001]	1.09 (1.08–1.1) [<0.0001]	1.09 (1.08–1.11) [<0.0001]	0.99 (0.98–1.01) [0.4212]
Risk homosexual vs. Heterosexual	0.96 (0.95–0.98) [<0.0001]	0.98 (0.96-1) [0.0199]	0.98 (0.97–0.99) [0.001]	0.97 (0.95–0.98) [<0.0001]	0.99 (0.97-1) [0.1498]
Risk intravenous drug user vs. Heterosexual	0.97 (0.95–0.99) [0.0008]	1.04 (1.02–1.07) [<0.0001]	0.96 (0.94–0.97) [<0.0001]	0.98 (0.96-1) [0.0432]	1.04 (1.02–1.06) [0.0002]
Risk mother-to-child vs. Heterosexual	1.03 (1-1.05) [0.0535]	1.11 (1.08–1.15) [<0.0001]	1.01 (0.99–1.03) [0.3205]	1.02 (1-1.05) [0.0576]	1.09 (1.06–1.13) [<0.0001]
Risk other/unknown vs. Heterosexual	1.09 (1.08–1.11) [<0.0001]	1.08 (1.07–1.1) [<0.0001]	1.05 (1.04–1.07) [<0.0001]	1.09 (1.08–1.11) [<0.0001]	1.02 (1.01–1.04) [0.0077]
Risk sex worker vs. Heterosexual	1.01 (0.97–1.05) [0.59]	1 (0.95–1.04) [0.8667]	1.01 (0.97–1.04) [0.6523]	1.01 (0.97–1.05) [0.5239]	0.99 (0.95–1.03) [0.5638]
Antiretroviral treatment-naïve	0.82 (0.81–0.82) [<0.0001]	0.83 (0.82–0.84) [<0.0001]	0.91 (0.9–0.92) [<0.0001]	0.83 (0.82–0.83) [<0.0001]	0.92 (0.91–0.93) [<0.0001]
Calendar year (per 10 years increase)	0.95 (0.94–0.96) [<0.0001]	1.03 (1.02–1.04) [<0.0001]	0.92 (0.91–0.92) [<0.0001]	0.97 (0.96–0.98) [<0.0001]	1.04 (1.02–1.06) [<0.0001]
Age <26 vs. 26 to 33	1.21 (1.18–1.24) [<0.0001]	1.07 (1.05–1.1) [<0.0001]	1.03 (1.01–1.05) [0.0022]	1.15 (1.13–1.18) [<0.0001]	1 (0.97–1.03) [0.9323]
Age 34 to 44 vs. 26 to 33	0.98 (0.96–1.01) [0.164]	0.98 (0.96–1.01) [0.1699]	0.98 (0.96-1) [0.0843]	0.98 (0.95-1) [0.0308]	1.04 (1.01–1.07) [0.0023]
Age >44 vs. 26 to 33	1 (0.97–1.03) [0.9696]	1 (0.97–1.03) [0.9638]	1.01 (0.98–1.03) [0.5096]	0.99 (0.97–1.02) [0.6363]	1.05 (1.03–1.08) [<0.0001]
Age unknown vs. 26 to 33	0.92 (0.9–0.94) [<0.0001]	0.89 (0.87–0.91) [<0.0001]	1.01 (1-1.03) [0.0957]	0.92 (0.9–0.94) [<0.0001]	0.93 (0.91–0.95) [<0.0001]
Subtype B vs. non-B/CRFs	1.14 (1.13–1.16) [<0.0001]	1.07 (1.06–1.08) [<0.0001]	1.08 (1.07–1.09) [<0.0001]	1.11 (1.1–1.12) [<0.0001]	1.02 (1.01–1.03) [0.0056]
Africa vs. Europe	0.94 (0.93–0.96) [<0.0001]	1.01 (0.99–1.02) [0.4348]	0.87 (0.85–0.88) [<0.0001]	0.95 (0.94–0.96) [<0.0001]	1.1 (1.08–1.12) [<0.0001]
Asia vs. Europe	0.88 (0.87–0.9) [<0.0001]	0.87 (0.86–0.88) [<0.0001]	0.91 (0.9–0.91) [<0.0001]	0.89 (0.88–0.9) [<0.0001]	0.96 (0.95–0.98) [<0.0001]
Caribbean vs. Europe	0.84 (0.8–0.88) [<0.0001]	0.88 (0.83–0.93) [<0.0001]	0.82 (0.79–0.86) [<0.0001]	0.83 (0.79–0.88) [<0.0001]	0.99 (0.94–1.05) [0.7716]
Central America vs. Europe	0.97 (0.92–1.02) [0.1789]	1.06 (1.01–1.12) [0.0185]	0.91 (0.88–0.95) [<0.0001]	1.02 (0.97–1.06) [0.4443]	1.4 (1.32–1.49) [<0.0001]
Russian Federation/USSR vs. Europe	0.91 (0.89–0.93) [<0.0001]	0.88 (0.86–0.91) [<0.0001]	0.9 (0.88–0.91) [<0.0001]	0.89 (0.87–0.91) [<0.0001]	1 (0.97–1.02) [0.7228]
Middle-East vs. Europe	0.86 (0.79–0.95) [0.0025]	0.81 (0.74–0.9) [<0.0001]	0.89 (0.82–0.96) [0.0022]	0.85 (0.77–0.93) [0.0002]	1.01 (0.9–1.13) [0.8985]
North America vs. Europe	0.98 (0.97-1) [0.0168]	0.92 (0.9–0.93) [<0.0001]	0.96 (0.95–0.98) [<0.0001]	0.95 (0.94–0.97) [<0.0001]	0.95 (0.93–0.97) [<0.0001]
Oceania vs. Europe	0.72 (0.69–0.74) [<0.0001]	0.76 (0.73–0.79) [<0.0001]	0.83 (0.8–0.85) [<0.0001]	0.72 (0.69–0.74) [<0.0001]	0.81 (0.78–0.85) [<0.0001]
South America vs. Europe	0.86 (0.84–0.88) [<0.0001]	0.87 (0.85–0.9) [<0.0001]	0.88 (0.87–0.9) [<0.0001]	0.86 (0.84–0.88) [<0.0001]	1 (0.98–1.03) [0.7959]

By continuing on Table 2, HIV-1 transmission by homosexual contact showed lower odds of drug resistance to any of the three inhibition class, and resistance to two or more classes (see also the association of homosexual transmission with B subtype). Injection drug users were at higher risk to carry drug resistance to NNRTIs but lower to other classes. Mother-to-child transmission was instead associated to higher risk of carrying any-class resistance. As expected, antiretroviral-naïve people had lower odds to present with drug resistance to NRTI/NNRTI/PIs as well as to multi-class. People younger than 26 years old had higher risk to carry a resistant virus to any drug class and multi-class as compared to older people.

In terms of continental areas, all had lower odds of drug resistance as compared to Europe (Table 2). We also performed a stratified analysis in the Los Alamos dataset looking at sequences coming from the same continental zone (Table S2). Overall, results were consistent with the main analysis (e.g., odds for non-B subtypes/CRFs, treatment-naïve, and risk groups). We found significant differences in the odds of having a resistant virus for patients belonging to different countries in the same continental zone.

Non-B subtypes/CRFs were associated to lower rates of drug resistance (Table 2); however, when breaking down the non-B subtypes/CRFs, some exhibited higher odds of drug resistance, in particular: 04_cpx, O and P for all classes; C and 49_cpx for NNRTIs; and 46_BF for PIs (Table S3). Of note, the kappa agreement between B subtype and antiretroviral-naïve was very poor, i.e., −0.06, with 50% of the sequences being both antiretroviral-naïve and non-B, or the opposite.

**Figure 2:**
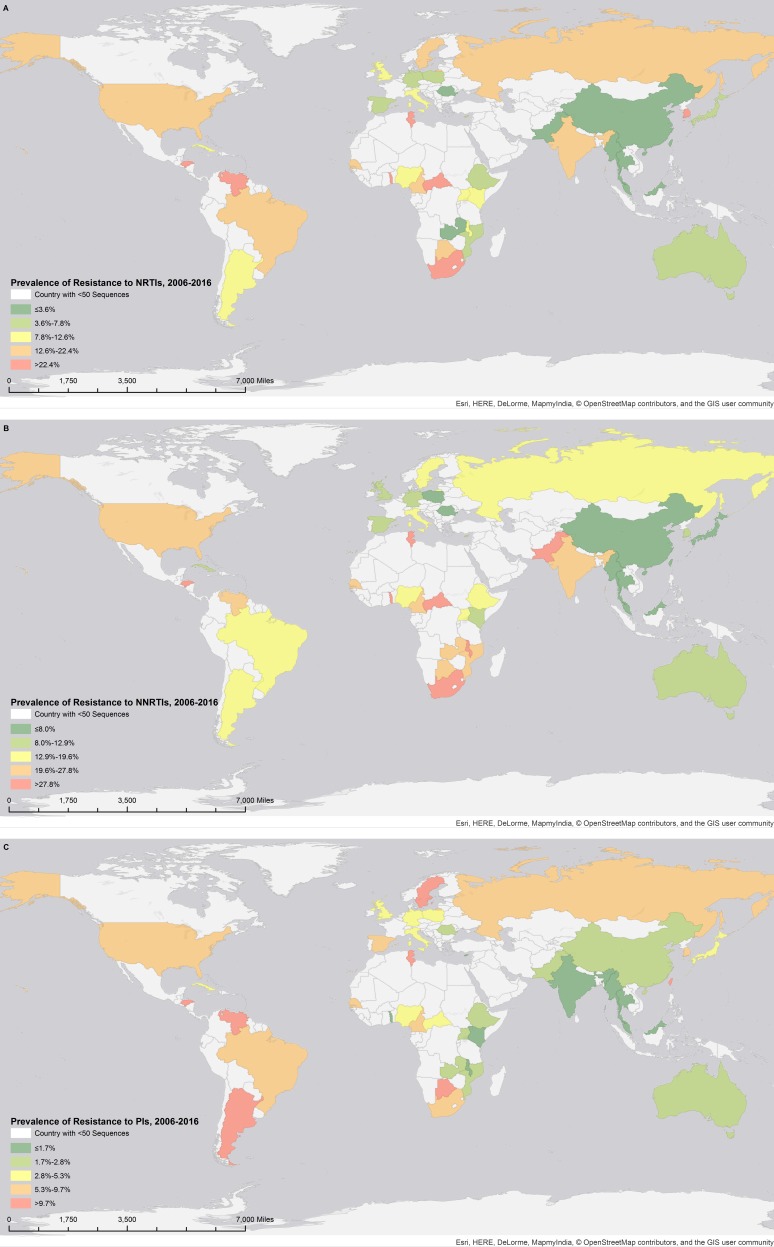
Prevalence of HIV drug resistance to NRTI (A), NNRTI (B), and PI (C) classes between 2006 and 2016. Image from OpenStreetMap, and the cartography is released under a CC-BY-SA license.

Then we passed on to analyzing data from the most recent decade (2006 to 2016). Figure 2 shows drug resistance prevalence to NRTIs, NNRTIs, and PIs for the countries included in the Los Alamos dataset (see Fig. S2 for multi-class resistance). By performing the same multivariable analysis, we found that a more recent calendar year was associated to an increased risk of resistance to any of these three drugs; isolates from Africa and Central America had also higher odds of presenting with drug resistance to new-generation inhibitors as compared to Europe.

We then looked at factors associated with a B vs. non-B subtype (Table 3). Male homosexuals were more likely to carry a B subtype than heterosexuals, whilst intravenous drug users had lower odds. There has been a substantial decrease (about 10%/year) in rate of B subtypes recorded in the data base, with higher odds of carrying non-B subtypes/CRFs for adults 44+ years old. Africa, Asia, the Caribbean, former USSR/Russian Federation, Middle-East, and South America isolates had lower odds of being B subtypes as compared to European isolates, whereas Central America, North America and Oceania were more likely to be B subtypes.

**Table 3:** Geodemographic factors associated with HIV-1 B vs. non-B subtype and circulating recombinant forms (CRFs).

Data attribute	Subtype B vs. non-B/CRFs Odds ratio (95% Confidence Interval) [*P*-value]
Sex M vs. F	1.09 (1.08–1.1)[<0.0001]
Sex unknown vs. F	1.09 (1.07–1.1)[<0.0001]
Risk homosexual vs. Heterosexual	1.18 (1.16–1.2)[<0.0001]
Risk intravenous drug user vs. Heterosexual	0.91 (0.9–0.93)[<0.0001]
Risk mother-to-child vs. Heterosexual	1.15 (1.12–1.18)[<0.0001]
Risk other/unknown vs. Heterosexual	1.1 (1.08–1.11)[<0.0001]
Risk sex worker vs. Heterosexual	0.96 (0.92–0.99)[0.0204]
Antiretroviral treatment-naïve	0.97 (0.96–0.98)[<0.0001]
Calendar year (per 10 years increase)	0.9 (0.89–0.91)[<0.0001]
Age <26 vs. 26 to 33	1.02 (0.99–1.04)[0.1293]
Age 34 to 44 vs. 26 to 33	0.98 (0.96–1.01)[0.1533]
Age >44 vs. 26 to 33	0.94 (0.91–0.96)[<0.0001]
Age unknown vs. 26 to 33	1.15 (1.13–1.17)[<0.0001]
Africa vs. Europe	0.52 (0.51–0.53)[<0.0001]
Asia vs. Europe	0.61 (0.6–0.61)[<0.0001]
Caribbean vs. Europe	0.76 (0.72–0.8)[<0.0001]
Central America vs. Europe	1.31 (1.25–1.37)[<0.0001]
Russian Federation/USSR vs. Europe	0.59 (0.58–0.6)[<0.0001]
Middle-East vs. Europe	0.66 (0.61–0.72)[<0.0001]
North America vs. Europe	1.03 (1.02–1.05)[<0.0001]
Oceania vs. Europe	1.13 (1.09–1.17)[<0.0001]
South America vs. Europe	0.93 (0.91–0.95)[<0.0001]

**Figure 3:**
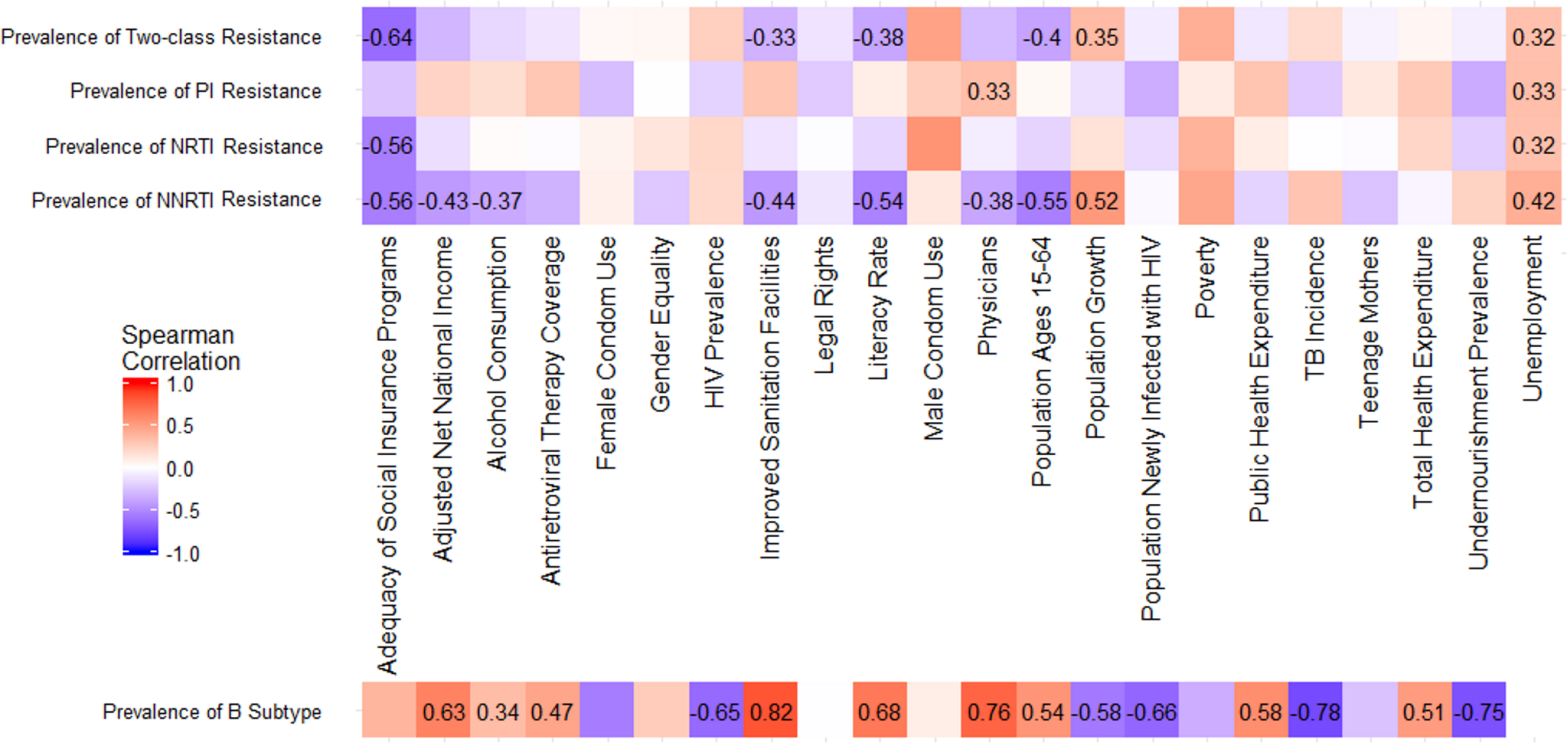
Heatmap showing correlation of drug resistance and subtype prevalence (country-by-country, 2006–2016) with sociodemographic indicators. Correlations values significant at the 5% level are shown.

With GIS analysis (Fig. 3), we found that higher rates of drug resistance were associated to unemployment, whilst lower rates were correlated with adequacy of social insurance programs. A higher prevalence of B subtype was correlated to improved sanitation facilities, physicians, higher literacy rates, adjusted net national income, and public health expenditure. Conversely, higher non-B/CRFs prevalence was correlated with high tuberculosis incidence, undernourishment, newly infected population, higher HIV prevalence, and population growth.

## Discussion

While development and circulation of HIV drug resistance is recognized as a relevant worldwide issue, estimating its burden and evolution is a challenging task. Information on drug resistance is commonly obtained through cohort studies or national and supranational HIV sequence repositories, sometimes created with different and specific aims and hence introducing sampling biases and failing to represent the whole patient population. In addition, the global view is seriously biased by a far larger number of studies done in high with respect to low/middle income countries with high HIV prevalence, two scenarios where drug availability and prescription policies have widely diverged for a long time. For example, a recent official document from the WHO has been based on 16 nationally representative surveys from only 14 low/middle income countries accounting for around 4,000 untreated and 300 treated patients with very limited representation from Southeast Asia (World Health Organization, 2017). The Los Alamos data base, even though it is not a formal surveillance medium, offers an opportunity to analyze a large number of sequences derived from a comprehensive variety of geographic areas. In addition, structured queries can be run to retrieve important information associated with HIV genotype. The overall worldwide trends of drug resistance obtained from Los Alamos data base in this work were confirmed by a separate analysis on the Stanford HIVdb Genotype-rx datasets, yet Los Alamos and HIVdb differ in terms of country, subtype distribution, and proportion of people on treatment.

Nonetheless, this study approach brings a number of limitations, in particular related to sampling bias. First, some countries are strongly over- or under-represented in these databases in relation to their HIV incidence. Second, different types of patients are sampled for different dates and countries. Third, for many sequences no information on prior treatment is available: this concern applies especially to sequences from treatment experienced patients (for which treatment history plays a key role in the observed resistance patterns) but to a lesser extent also for treatment naive sequences. As a general note of caution, since the prevalence of drug resistance in untreated vs. treated patients differs considerably, any global estimate must correct available data for the rate of antiretroviral coverage in specific geographic areas.

Standing such challenges associated with interpreting sequence data for which metadata is not uniformly available, with a careful, stratified study design is still possible to draw estimates that can be corroborated by independent sources. In fact, our study capitalizes on the meta-analysis of Rhee et al. (2015) up on which Stanford HIVdb provides freely a large sequence selection and curation, and moves forward by looking at individual demographics and country-specific social-ecological determinants by GIS analysis. Therefore, large-scale studies like ours can be carried out in parallel with more traditional approaches that may suffer from small sample size, or meta-analyses that increase the sample size but necessarily discard a number of attributes.

A major finding of this work is the divergent temporal trends in drug resistance with B vs. non-B subtypes and CRFs. Although not strictly, these categories tend to represent high- and low/middle-income countries, respectively. While an increase in drug resistance is reasonably expected along with ART rollout in resource constrained areas, the plateau effect in decreasing drug resistance in areas covered by the most modern drug regimens warns against the downgrading of the resistance issue in Western countries, a vision supported by some large size studies published a few years ago. Indeed, although potent and well tolerated, mostly high genetic barrier regimens have been increasingly used, novel drug classes have been not made available since 2008, suggesting that cross-resistance may play a major role in carrying forward a proportion of patients with detectable viremia still fueling drug resistance at population level. Increasing resistance to latest drugs in more recent years, particularly those with high genetic barrier such as darunavir and etravirine, is indeed consistent with expanded use of these drugs but also with cumulative exposure to previously available cross-resistant drugs.

On the other hand, transmitted drug resistance appears to remain stable over time. Notably, stable primary resistance rates have been observed even in areas where emergent resistance has declined considerably, likely due to a process of selection whereby patients failing therapy and harboring drug-resistant virus are favored over patients with undetectable viremia and wild type virus in terms of HIV transmission. However, large clusters of wild type virus transmission are maintained via unsafe practices among individuals unaware of their HIV status or not yet under treatment (Olson et al., 2018).

It must be noted that adherence clearly plays an important role in determining the rate of emergent resistance. A higher prevalence of drug resistance was indeed observed in intravenous drug users with respect to other risk groups but only for NNRTI. The most widely used drugs in this class are well known for their long half-life and low genetic barrier resulting in functional monotherapy and ease of selection of drug resistance upon erratic adherence, a behavior often associated with intravenous drug use. On the other hand, very poor adherence may have also been responsible for the association between lower rates of resistance and healthcare indicators as well as literacy rate. In regard to the correlation of subtype with a number of social-ecological determinants, e.g., sanitation, physicians, public health expenditure, this in part reflects the known fact that subtype B is the predominant subtype in the US and western Europe. However, many of these sociodemographic indices also vary considerably among countries with majority of non-B subtypes, and our findings warrant a deepened analysis that may help public health officials to strategies welfare interventions.

The large sample size of this study allowed for comparisons between B and individual non-B subtypes/CRFs, rather than between B and non-B subtypes/CRFs, a convenience grouping commonly used but which does not reflect any biologically significant category. Apart from a small increase in NNRTI resistance with subtype C, we did not observe relevant differences, suggesting that resistance currently impacts different subtypes/CRFs with comparable rates, a finding also in agreement with the lack of evidence of differential outcome of ART with different HIV clades. The high levels of resistance to some rare recombinants (i.e., CRF04_cpx) may be probably due to the low number of sequences available. Therefore for rare variants the findings may be heavily biased.

An important limitation of this study is that resistance to integrase inhibitors was not analyzed. While this drug class is expected to be dominant in ART, both in high and low/middle income countries, currently scoring algorithms and isolate data in Los Alamos data base do not allow deriving robust estimates; Stanford HIVdb provides a larger collection of sequences, but the individual-level relevant demographic information is not available. However, updates are warranted along with increasing collection of integrase sequences over time. It is expected that the introduction of high genetic barrier integrase inhibitors such as dolutegravir, cabotegravir and bictegravir will limit emergence and transmission of resistance to this drug class.

Given the diverse collection policies and sources of sequence provenience for the Los Alamos and Stanford HIVdb repositories, we are aware that a proper sample selection for minimizing bias is difficult, and some of the findings here reported may have been affected by this issue.

## Conclusions

Data obtained from large and in part curated collections of HIV sequences can effectively complement resistance centered survey such as those reported by the WHO to derive the global burden of drug resistance and inform future surveillance and education programs.

##  Supplemental Information

10.7717/peerj.4848/supp-1 Supplemental Information 1 Genbank accession numbers List of Genbank accession numbers used in the analysis.

10.7717/peerj.4848/supp-2 Supplemental Information 2 Supplementary Material

## Additional Information and Declarations

### Data Availability

The following information was supplied regarding data availability: HIV pol sequences were downloaded from the following public repositories: (i) Los Alamos HIV data bases, https://www.hiv.lanl.gov/content/sequence/HIV/mainpage.html; and (ii) Stanford HIVdb, https://hivdb.stanford.edu/pages/geno-rx-datasets.html. The GenBank’s accession numbers for the raw data are provided in the Supplemental File.
