# Supplementary material for: The global burden of HIV-1 drug resistance in the past 20 years
Source: PeerJ. 2018 May 25;6:e4848. doi: 10.7717/peerj.4848 (PMC5971836; doi:10.7717/peerj.4848)
Supplement: Supplemental Information 2 [file peerj-06-4848-s002.docx]

**Supplementary Material**

**Supplementary Figure S1.** HIVdb data base. Prevalence of NRTI, NNRTI, PI, two-class, DRV/ETR/RPV, and transmitted drug resistance (in therapy-naïve people) by calendar year and by B vs. non-B subtypes and circulating recombinant forms (CRFs). Point estimates indicate per-year prevalence, whilst line estimates are drawn by lowess interpolation and data bootstrapping (150 times).


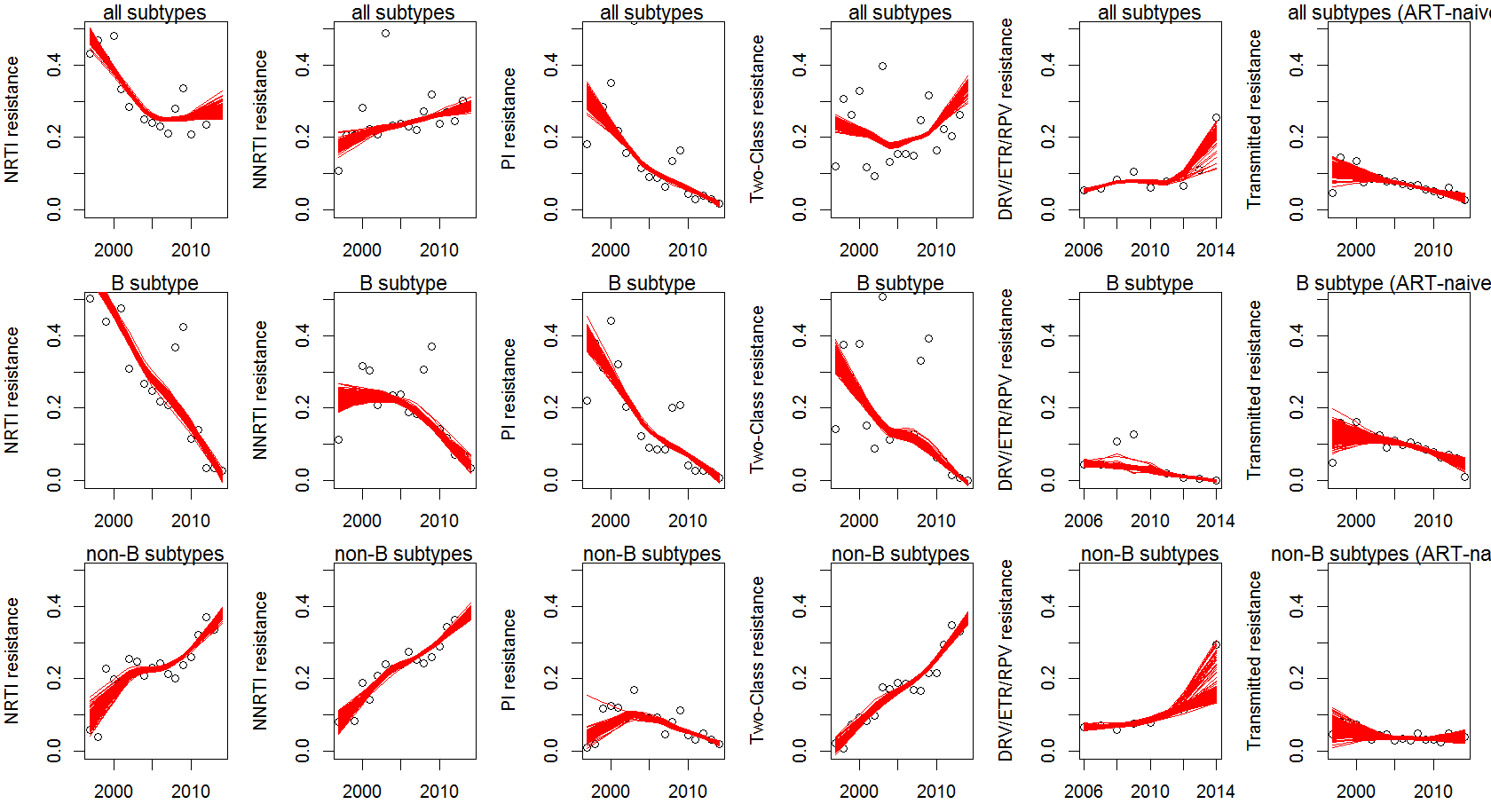


**Supplementary Figure S2.** Prevalence of two-class (at least two among NRTI/NNRTI/PI) HIV drug resistance between 2006 and 2016 (Los Alamos data base).

**
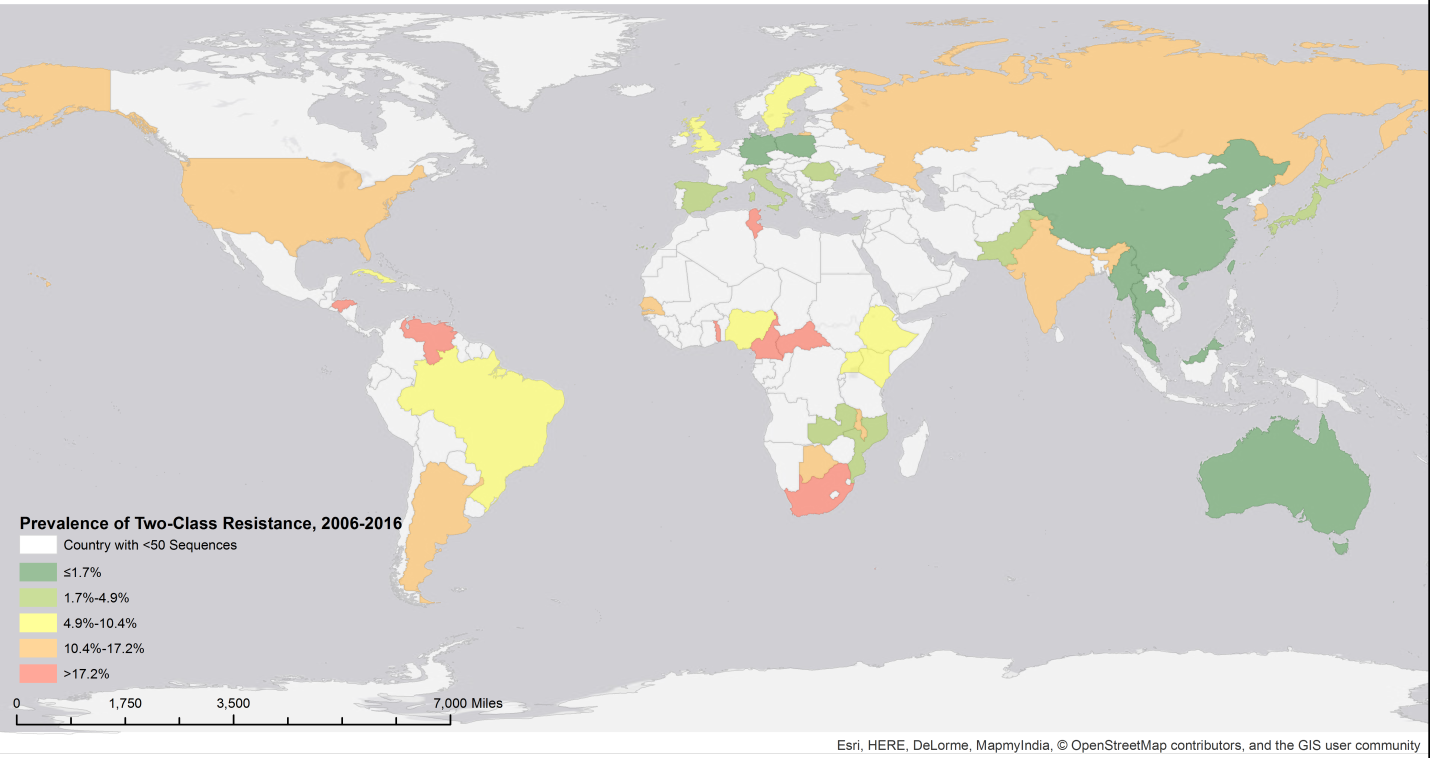
**

**Supplementary Table S1.** Rates of agreement (Cohen’s kappa) between HIVdb, Rega and ANRS in scoring NRTI/NNRTI/PI resistance, stratified by B vs. non-B subtypes and circulating recombinant forms (CRFs).

| NRTI/NNRTI/PI | B subtype | | Non-B subtypes/CRFs | |
| --- | --- | --- | --- | --- |
|  | HIVdb | Rega | HIVdb | Rega |
| Rega | 0.97/0.95/0.90 |  | 0.96/0.97/0.75 |  |
| ANRS | 0.96/0.81/0.90 | 0.97/0.83/0.90 | 0.97/0.92/0.01 | 0.97/0.94/0.01 |

**Supplementary Table S2.** Multivariable logistic regression on the drug resistance outcome (NRTI/NNRTI/PI) stratified by continental area.

| Continental Area | Data Attribute / Resistance To Drug Class | NRTI | NNRTI | PI |
| --- | --- | --- | --- | --- |
|  |  | Odds Ratio (95% Confidence Interval) [P-Value] | | |
| Africa | sex M vs. F | 1.04 (1.02-1.06) [0.0005] | 1 (0.98-1.03) [0.7028] | 1.01 (0.99-1.02) [0.4034] |
|  | sex UNK vs. F | 1.01 (0.99-1.03) [0.2362] | 0.91 (0.89-0.94) [<0.0001] | 1.02 (1.01-1.03) [0.0008] |
|  | risk Homosexual vs. Heterosexual | 0.89 (0.84-0.94) [<0.0001] | 0.92 (0.86-0.98) [0.0157] | 0.98 (0.95-1.01) [0.2078] |
|  | risk MotherToChild vs. Heterosexual | 0.99 (0.95-1.03) [0.531] | 1.21 (1.15-1.27) [<0.0001] | 1 (0.97-1.02) [0.7937] |
|  | risk OtherUnknown vs. Heterosexual | 1.1 (1.07-1.13) [<0.0001] | 1.16 (1.13-1.2) [<0.0001] | 1 (0.99-1.02) [0.7728] |
|  | risk SexWorker vs. Heterosexual | 0.87 (0.79-0.96) [0.0045] | 0.81 (0.72-0.9) [0.0002] | 0.97 (0.91-1.03) [0.2783] |
|  | Antiretroviral Treatment-Naïve | 0.82 (0.81-0.84) [<0.0001] | 0.78 (0.77-0.8) [<0.0001] | 0.98 (0.97-1) [0.0096] |
|  | Calendar Year (per 10 years increase) | 1.01 (1.01-1.02) [<0.0001] | 1.01 (1.01-1.01) [<0.0001] | 1 (1-1) [0.8287] |
|  | age 34 to 44 vs. 26 to 33 | 1.01 (0.97-1.05) [0.6024] | 1.02 (0.97-1.06) [0.428] | 1.03 (1-1.05) [0.0336] |
|  | age above 44 vs. 26 to 33 | 1.04 (0.99-1.09) [0.1179] | 1.04 (0.98-1.09) [0.2288] | 1.05 (1.02-1.08) [0.0015] |
|  | age below 26 vs. 26 to 33 | 1.21 (1.16-1.25) [<0.0001] | 1.01 (0.97-1.06) [0.5317] | 1.03 (1.01-1.05) [0.0159] |
|  | age unknown vs. 26 to 33 | 0.87 (0.84-0.9) [<0.0001] | 0.88 (0.85-0.92) [<0.0001] | 1.01 (0.99-1.03) [0.2063] |
|  | subtype B vs. non-B/CRFs | 0.98 (0.92-1.05) [0.5808] | 0.9 (0.84-0.97) [0.0086] | 1.11 (1.07-1.15) [<0.0001] |
|  | BOTSWANA vs. South Africa | 0.81 (0.78-0.84) [<0.0001] | 0.79 (0.76-0.82) [<0.0001] | 1.09 (1.07-1.11) [<0.0001] |
|  | CAMEROON vs. South Africa | 0.82 (0.79-0.85) [<0.0001] | 0.81 (0.78-0.85) [<0.0001] | 1.02 (1-1.04) [0.1069] |
|  | OTHER vs. South Africa | 0.86 (0.85-0.88) [<0.0001] | 0.9 (0.88-0.92) [<0.0001] | 0.98 (0.97-0.99) [0.0003] |
|  | UGANDA vs. South Africa | 0.87 (0.85-0.89) [<0.0001] | 0.92 (0.89-0.95) [<0.0001] | 0.97 (0.95-0.98) [<0.0001] |
|  | ZAMBIA vs. South Africa | 0.93 (0.9-0.96) [<0.0001] | 1.04 (1-1.08) [0.0575] | 0.98 (0.96-1) [0.0366] |
| Asia / Oceania | sex M vs. F | 1.01 (0.99-1.03) [0.2171] | 1 (0.98-1.02) [0.8388] | 1 (0.99-1.01) [0.948] |
|  | sex UNK vs. F | 1.02 (1-1.04) [0.0226] | 1.05 (1.03-1.08) [<0.0001] | 1.02 (1-1.03) [0.0105] |
|  | risk Homosexual vs. Heterosexual | 0.96 (0.95-0.98) [<0.0001] | 0.96 (0.94-0.98) [0.0002] | 1.01 (1-1.02) [0.2267] |
|  | risk IVDrugUser vs. Heterosexual | 0.96 (0.94-0.98) [<0.0001] | 1.01 (0.99-1.03) [0.4267] | 0.98 (0.96-0.99) [0.0009] |
|  | risk MotherToChild vs. Heterosexual | 1.13 (1.05-1.21) [0.001] | 1.3 (1.19-1.41) [<0.0001] | 1.09 (1.03-1.15) [0.0023] |
|  | risk OtherUnknown vs. Heterosexual | 1 (0.98-1.02) [0.7415] | 0.95 (0.93-0.97) [<0.0001] | 1 (0.98-1.01) [0.7461] |
|  | risk SexWorker vs. Heterosexual | 0.98 (0.94-1.02) [0.2334] | 0.96 (0.92-1.01) [0.1067] | 0.99 (0.96-1.02) [0.6634] |
|  | Antiretroviral Treatment-Naïve | 0.95 (0.94-0.96) [<0.0001] | 0.94 (0.93-0.95) [<0.0001] | 1.01 (1-1.01) [0.1064] |
|  | Calendar Year (per 10 years increase) | 1 (1-1) [0.738] | 1 (1-1.01) [0.0007] | 1 (1-1) [0.0052] |
|  | age 34 to 44 vs. 26 to 33 | 1 (0.97-1.03) [0.9555] | 0.97 (0.93-1.01) [0.1339] | 0.98 (0.95-1) [0.0849] |
|  | age above 44 vs. 26 to 33 | 0.99 (0.95-1.04) [0.6899] | 0.98 (0.93-1.03) [0.3807] | 1.01 (0.97-1.04) [0.7582] |
|  | age below 26 vs. 26 to 33 | 1 (0.97-1.04) [0.8298] | 1.01 (0.97-1.06) [0.5155] | 1.03 (1-1.06) [0.0259] |
|  | age unknown vs. 26 to 33 | 0.98 (0.95-1) [0.0548] | 0.94 (0.91-0.97) [<0.0001] | 0.99 (0.97-1.01) [0.5632] |
|  | subtype B vs. non-B/CRFs | 1.06 (1.04-1.07) [<0.0001] | 1.01 (0.99-1.03) [0.1902] | 1.01 (1-1.02) [0.271] |
|  | AUSTRALIA vs. China | 1 (0.96-1.03) [0.7547] | 1.11 (1.07-1.15) [<0.0001] | 1.05 (1.02-1.08) [0.0001] |
|  | INDIA vs. China | 1.12 (1.1-1.15) [<0.0001] | 1.23 (1.2-1.26) [<0.0001] | 0.99 (0.98-1.01) [0.5303] |
|  | JAPAN vs. China | 0.97 (0.95-1) [0.0199] | 0.99 (0.96-1.02) [0.4842] | 1.04 (1.02-1.06) [<0.0001] |
|  | OTHER vs. China | 1.03 (1.01-1.04) [0.0003] | 1.08 (1.06-1.1) [<0.0001] | 1.02 (1.01-1.03) [0.0005] |
|  | THAILAND vs. China | 1 (0.97-1.03) [0.784] | 1.03 (0.99-1.07) [0.1685] | 0.99 (0.97-1.02) [0.6663] |
| Central / South  America and  Caribbean | sex M vs. F | 1.07 (0.98-1.17) [0.1198] | 1 (0.91-1.1) [0.9789] | 1.04 (0.97-1.12) [0.3008] |
|  | sex UNK vs. F | 1.06 (0.98-1.13) [0.1393] | 1.02 (0.95-1.1) [0.534] | 0.99 (0.94-1.06) [0.8569] |
|  | risk Homosexual vs. Heterosexual | 0.99 (0.89-1.1) [0.8789] | 1.03 (0.92-1.15) [0.5876] | 0.99 (0.9-1.08) [0.791] |
|  | risk IVDrugUser vs. Heterosexual | 0.89 (0.72-1.09) [0.2444] | 0.97 (0.78-1.19) [0.7476] | 0.99 (0.83-1.17) [0.8652] |
|  | risk MotherToChild vs. Heterosexual | 0.93 (0.85-1.02) [0.119] | 0.95 (0.86-1.04) [0.2851] | 1.04 (0.97-1.13) [0.2746] |
|  | risk OtherUnknown vs. Heterosexual | 1.04 (0.96-1.12) [0.3688] | 0.99 (0.91-1.07) [0.7429] | 1.05 (0.98-1.12) [0.1748] |
|  | risk SexWorker vs. Heterosexual | 1.02 (0.88-1.17) [0.8355] | 1.1 (0.95-1.28) [0.186] | 1 (0.88-1.12) [0.9446] |
|  | Antiretroviral Treatment-Naïve | 0.87 (0.83-0.92) [<0.0001] | 0.91 (0.86-0.96) [0.0004] | 0.94 (0.9-0.98) [0.0085] |
|  | Calendar Year (per 10 years increase) | 1 (0.99-1.01) [0.9639] | 1.01 (1-1.02) [0.0097] | 1 (1-1.01) [0.4446] |
|  | age 34 to 44 vs. 26 to 33 | 0.85 (0.76-0.97) [0.0127] | 0.85 (0.75-0.97) [0.0156] | 0.84 (0.76-0.93) [0.0009] |
|  | age above 44 vs. 26 to 33 | 1.02 (0.86-1.21) [0.7772] | 1.12 (0.94-1.34) [0.1874] | 0.97 (0.84-1.12) [0.6988] |
|  | age below 26 vs. 26 to 33 | 1.07 (0.93-1.22) [0.3499] | 1.09 (0.95-1.26) [0.2058] | 0.89 (0.79-1) [0.0444] |
|  | age unknown vs. 26 to 33 | 0.97 (0.87-1.08) [0.5976] | 0.94 (0.84-1.06) [0.3065] | 0.93 (0.85-1.02) [0.1308] |
|  | subtype B vs. non-B/CRFs | 0.99 (0.94-1.04) [0.6604] | 0.98 (0.93-1.03) [0.4321] | 1 (0.96-1.04) [0.9045] |
|  | ARGENTINA vs. Brazil | 0.97 (0.9-1.06) [0.5076] | 0.98 (0.9-1.07) [0.7097] | 1.01 (0.94-1.08) [0.7448] |
|  | CUBA vs. Brazil | 0.99 (0.92-1.06) [0.7356] | 1.01 (0.94-1.1) [0.7249] | 0.98 (0.92-1.04) [0.4631] |
|  | HONDURAS vs. Brazil | 1.47 (1.34-1.62) [<0.0001] | 1.52 (1.38-1.67) [<0.0001] | 1.24 (1.15-1.34) [<0.0001] |
|  | OTHER vs. Brazil | 0.99 (0.93-1.07) [0.8668] | 1.04 (0.97-1.12) [0.2473] | 0.98 (0.93-1.04) [0.5222] |
|  | VENEZUELA vs. Brazil | 1.03 (0.97-1.11) [0.3245] | 1.06 (0.99-1.14) [0.1111] | 1.09 (1.03-1.15) [0.0027] |
| Europe /  Middle East /  Former USSR  and  Russian  Federation | sex M vs. F | 1 (0.97-1.03) [0.8067] | 0.97 (0.94-1) [0.0827] | 0.95 (0.92-0.97) [0.0001] |
|  | sex UNK vs. F | 1.27 (1.23-1.31) [<0.0001] | 1.18 (1.14-1.23) [<0.0001] | 1.22 (1.19-1.26) [<0.0001] |
|  | risk Homosexual vs. Heterosexual | 0.93 (0.9-0.96) [<0.0001] | 0.96 (0.93-1) [0.0258] | 0.96 (0.93-0.99) [0.0113] |
|  | risk IVDrugUser vs. Heterosexual | 0.97 (0.94-1.01) [0.1228] | 1 (0.96-1.04) [0.869] | 1.02 (0.98-1.05) [0.3103] |
|  | risk MotherToChild vs. Heterosexual | 1.03 (0.95-1.12) [0.4264] | 0.99 (0.91-1.08) [0.8469] | 0.94 (0.87-1.01) [0.0733] |
|  | risk OtherUnknown vs. Heterosexual | 1.01 (0.98-1.04) [0.647] | 1.03 (1-1.07) [0.0465] | 1.06 (1.03-1.09) [<0.0001] |
|  | risk SexWorker vs. Heterosexual | 1.02 (0.82-1.28) [0.8403] | 0.88 (0.69-1.13) [0.3216] | 0.94 (0.76-1.15) [0.521] |
|  | Antiretroviral Treatment-Naïve | 0.74 (0.72-0.75) [<0.0001] | 0.77 (0.76-0.79) [<0.0001] | 0.79 (0.77-0.8) [<0.0001] |
|  | Calendar Year (per 10 years increase) | 0.98 (0.98-0.98) [<0.0001] | 0.99 (0.99-1) [<0.0001] | 0.99 (0.98-0.99) [<0.0001] |
|  | age 34 to 44 vs. 26 to 33 | 1.03 (0.98-1.09) [0.2677] | 0.95 (0.89-1.01) [0.0765] | 0.99 (0.94-1.05) [0.8145] |
|  | age above 44 vs. 26 to 33 | 1 (0.93-1.07) [0.9754] | 0.96 (0.89-1.03) [0.2228] | 0.98 (0.92-1.04) [0.4452] |
|  | age below 26 vs. 26 to 33 | 1.05 (0.98-1.13) [0.1845] | 0.97 (0.89-1.05) [0.4053] | 1.04 (0.97-1.12) [0.2232] |
|  | age unknown vs. 26 to 33 | 0.95 (0.91-1) [0.0586] | 0.89 (0.84-0.94) [<0.0001] | 0.93 (0.89-0.97) [0.0009] |
|  | subtype B vs. non-B/CRFs | 1.22 (1.2-1.24) [<0.0001] | 1.14 (1.12-1.17) [<0.0001] | 1.22 (1.2-1.24) [<0.0001] |
|  | OTHER vs. Germany | 0.94 (0.91-0.97) [<0.0001] | 0.92 (0.9-0.95) [<0.0001] | 0.92 (0.9-0.95) [<0.0001] |
|  | POLAND vs. Germany | 1.04 (1.01-1.07) [0.0097] | 0.98 (0.95-1.01) [0.1584] | 1.01 (0.98-1.04) [0.5744] |
|  | RUSSIAN FEDERATION vs. Germany | 0.94 (0.9-0.97) [0.0004] | 0.89 (0.86-0.93) [<0.0001] | 0.86 (0.83-0.89) [<0.0001] |
|  | SPAIN vs. Germany | 0.84 (0.82-0.87) [<0.0001] | 0.85 (0.82-0.88) [<0.0001] | 0.85 (0.82-0.87) [<0.0001] |
|  | UNITED KINGDOM vs. Germany | 0.83 (0.81-0.85) [<0.0001] | 0.83 (0.81-0.86) [<0.0001] | 0.82 (0.8-0.84) [<0.0001] |
| North America | sex M vs. F | 1 (0.95-1.05) [0.8939] | 0.99 (0.94-1.05) [0.8299] | 1.02 (0.97-1.07) [0.5412] |
|  | sex UNK vs. F | 1.33 (1.24-1.43) [<0.0001] | 1.14 (1.06-1.23) [0.0004] | 1.22 (1.14-1.31) [<0.0001] |
|  | risk Homosexual | 1.07 (0.95-1.19) [0.2658] | 0.98 (0.87-1.1) [0.7183] | 1.08 (0.97-1.21) [0.1665] |
|  | risk IVDrugUser | 1.23 (1.04-1.46) [0.0172] | 0.98 (0.82-1.18) [0.822] | 1.13 (0.96-1.34) [0.1443] |
|  | risk MotherToChild | 1.33 (1.08-1.65) [0.0076] | 0.87 (0.7-1.1) [0.2452] | 1.15 (0.93-1.41) [0.1886] |
|  | risk OtherUnknown | 1.17 (1.04-1.3) [0.0068] | 0.94 (0.83-1.06) [0.304] | 1.15 (1.03-1.28) [0.0125] |
|  | Antiretroviral Treatment-Naïve | 0.88 (0.82-0.94) [<0.0001] | 1.04 (0.97-1.11) [0.3163] | 0.94 (0.88-1) [0.045] |
|  | Calendar Year (per 10 years increase) | 0.98 (0.97-0.98) [<0.0001] | 1 (0.99-1) [0.2835] | 0.98 (0.98-0.99) [<0.0001] |
|  | age 34 to 44 vs. 26 to 33 | 1.02 (0.95-1.1) [0.517] | 1.04 (0.96-1.12) [0.3249] | 1.02 (0.95-1.09) [0.6327] |
|  | age above 44 vs. 26 to 33 | 1.12 (1.04-1.21) [0.0034] | 1.06 (0.98-1.15) [0.163] | 1.07 (0.99-1.15) [0.0701] |
|  | age below 26 vs. 26 to 33 | 1.09 (1-1.19) [0.0392] | 1.03 (0.94-1.12) [0.563] | 1.06 (0.98-1.15) [0.1604] |
|  | age unknown vs. 26 to 33 | 1.01 (0.93-1.09) [0.8804] | 0.98 (0.9-1.07) [0.6356] | 1.05 (0.96-1.13) [0.2779] |
|  | subtype B vs. non-B/CRFs | 1.07 (1-1.15) [0.043] | 1 (0.93-1.08) [0.9922] | 1.02 (0.96-1.09) [0.5113] |
|  | CANADA vs. USA | 0.9 (0.79-1.02) [0.1041] | 0.91 (0.79-1.05) [0.1866] | 0.92 (0.81-1.04) [0.1991] |

**Supplementary Table S3.** Non-B subtypes and circulating recombinant forms (CRFs) associated with genotypic resistance to HIV-1 NRTIs, NNRTIs, PIs (models are adjusted for geodemographic factors).

| Individual non-B subtype/CRF  (vs. B subtype) | N | NRTI | NNRTI | PI |
| --- | --- | --- | --- | --- |
|  |  | Odds Ratio (95% Confidence Interval) [P-Value] | | |
| 01_AE | 3911 | 0.92 (0.9-0.93) [<0.0001] | 0.93 (0.92-0.95) [<0.0001] | 0.98 (0.96-0.99) [0.0002] |
| 02_AG | 1225 | 0.8 (0.78-0.82) [<0.0001] | 0.91 (0.89-0.93) [<0.0001] | 0.85 (0.84-0.87) [<0.0001] |
| 03_AB | 25 | 0.85 (0.74-0.97) [0.0197] | 0.86 (0.74-1) [0.055] | 0.91 (0.81-1.02) [0.0907] |
| 04_cpx | 10 | 1.55 (1.24-1.92) [<0.0001] | 1.44 (1.14-1.82) [0.0025] | 1.48 (1.24-1.76) [<0.0001] |
| 05_DF | 6 | 0.61 (0.46-0.8) [0.0004] | 0.68 (0.5-0.92) [0.0119] | 0.77 (0.62-0.97) [0.0269] |
| 06_cpx | 124 | 0.87 (0.82-0.93) [<0.0001] | 0.88 (0.82-0.94) [0.0003] | 0.89 (0.85-0.94) [<0.0001] |
| 07_BC | 1720 | 0.95 (0.93-0.97) [<0.0001] | 0.93 (0.91-0.95) [<0.0001] | 0.99 (0.98-1.01) [0.4952] |
| 08_BC | 476 | 0.87 (0.84-0.9) [<0.0001] | 0.93 (0.9-0.97) [0.0002] | 0.92 (0.89-0.94) [<0.0001] |
| 09_cpx | 26 | 0.76 (0.66-0.87) [<0.0001] | 0.83 (0.72-0.96) [0.0116] | 0.93 (0.83-1.03) [0.1743] |
| 10_CD | 27 | 0.75 (0.65-0.85) [<0.0001] | 0.79 (0.68-0.91) [0.0011] | 0.84 (0.76-0.94) [0.0016] |
| 11_cpx | 94 | 0.81 (0.75-0.87) [<0.0001] | 0.9 (0.83-0.97) [0.0062] | 0.89 (0.84-0.94) [0.0001] |
| 12_BF | 119 | 0.89 (0.83-0.95) [0.0003] | 0.96 (0.9-1.03) [0.2707] | 0.92 (0.88-0.97) [0.003] |
| 13_cpx | 17 | 0.93 (0.78-1.09) [0.3653] | 0.94 (0.78-1.12) [0.4873] | 1.08 (0.94-1.23) [0.2719] |
| 14_BG | 155 | 0.87 (0.82-0.92) [<0.0001] | 0.91 (0.85-0.96) [0.0013] | 0.94 (0.9-0.98) [0.0053] |
| 15_01B | 1045 | 0.9 (0.88-0.92) [<0.0001] | 0.92 (0.9-0.95) [<0.0001] | 0.94 (0.92-0.96) [<0.0001] |
| 16_A2D | 16 | 0.96 (0.81-1.14) [0.6756] | 0.83 (0.69-1) [0.0545] | 0.91 (0.79-1.04) [0.1751] |
| 17_BF | 43 | 0.91 (0.82-1.01) [0.0739] | 1.1 (0.98-1.23) [0.0996] | 0.95 (0.87-1.03) [0.2019] |
| 18_cpx | 31 | 0.86 (0.76-0.98) [0.0214] | 1.09 (0.95-1.25) [0.227] | 0.91 (0.82-1) [0.0606] |
| 19_cpx | 48 | 1 (0.9-1.11) [0.9652] | 0.99 (0.89-1.11) [0.9067] | 1.04 (0.96-1.13) [0.3335] |
| 20_BG | 34 | 0.91 (0.81-1.03) [0.1352] | 0.95 (0.83-1.08) [0.4347] | 0.94 (0.85-1.04) [0.2332] |
| 21_A2D | 10 | 0.73 (0.59-0.91) [0.0045] | 0.79 (0.63-1.01) [0.0553] | 0.8 (0.67-0.95) [0.0108] |
| 22_01A1 | 240 | 0.86 (0.82-0.9) [<0.0001] | 0.93 (0.89-0.98) [0.0059] | 0.89 (0.86-0.93) [<0.0001] |
| 23_BG | 8 | 1.25 (0.98-1.6) [0.0748] | 0.98 (0.75-1.27) [0.8591] | 1.17 (0.96-1.43) [0.1137] |
| 24_BG | 27 | 0.85 (0.74-0.98) [0.0269] | 0.85 (0.73-0.99) [0.0359] | 0.92 (0.82-1.03) [0.1291] |
| 25_cpx | 28 | 0.83 (0.73-0.94) [0.0045] | 0.94 (0.82-1.09) [0.422] | 0.84 (0.76-0.94) [0.0015] |
| 26_AU | 11 | 0.68 (0.56-0.84) [0.0003] | 0.84 (0.67-1.06) [0.1401] | 0.84 (0.71-0.99) [0.0424] |
| 27_cpx | 5 | 0.6 (0.44-0.81) [0.001] | 1.22 (0.87-1.7) [0.2414] | 0.76 (0.6-0.98) [0.0329] |
| 28_BF | 50 | 0.98 (0.89-1.08) [0.7425] | 0.97 (0.87-1.08) [0.577] | 1.01 (0.94-1.1) [0.7239] |
| 29_BF | 21 | 1.02 (0.88-1.19) [0.7686] | 1.15 (0.97-1.35) [0.0989] | 0.99 (0.88-1.12) [0.931] |
| 31_BC | 155 | 0.86 (0.81-0.91) [<0.0001] | 1.02 (0.96-1.08) [0.5898] | 0.9 (0.86-0.94) [<0.0001] |
| 32_06A1 | 12 | 0.72 (0.59-0.87) [0.001] | 0.83 (0.67-1.03) [0.0973] | 0.82 (0.7-0.97) [0.0183] |
| 33_01B | 126 | 0.82 (0.76-0.87) [<0.0001] | 0.83 (0.77-0.89) [<0.0001] | 0.9 (0.85-0.94) [<0.0001] |
| 34_01B | 13 | 0.9 (0.74-1.09) [0.2691] | 0.89 (0.72-1.1) [0.2769] | 0.94 (0.8-1.09) [0.4013] |
| 35_AD | 65 | 0.82 (0.75-0.9) [<0.0001] | 0.93 (0.85-1.03) [0.1916] | 0.87 (0.81-0.94) [0.0003] |
| 36_cpx | 73 | 0.77 (0.71-0.83) [<0.0001] | 0.87 (0.79-0.95) [0.0014] | 0.87 (0.81-0.93) [<0.0001] |
| 37_cpx | 8 | 0.71 (0.56-0.91) [0.0057] | 0.86 (0.66-1.11) [0.2457] | 0.83 (0.68-1.01) [0.0651] |
| 38_BF1 | 7 | 0.97 (0.75-1.26) [0.8097] | 0.86 (0.65-1.14) [0.3004] | 0.94 (0.76-1.15) [0.5319] |
| 39_BF | 2 | 1.26 (0.78-2.05) [0.3419] | 1.37 (0.81-2.31) [0.2452] | 1.33 (0.9-1.97) [0.1572] |
| 40_BF | 2 | 1.13 (0.7-1.83) [0.623] | 1.25 (0.74-2.12) [0.4] | 1.24 (0.83-1.83) [0.2908] |
| 42_BF | 43 | 0.59 (0.53-0.66) [<0.0001] | 0.68 (0.6-0.76) [<0.0001] | 0.68 (0.62-0.74) [<0.0001] |
| 43_02G | 68 | 0.8 (0.74-0.88) [<0.0001] | 0.88 (0.8-0.96) [0.0049] | 0.82 (0.77-0.88) [<0.0001] |
| 44_BF | 3 | 0.63 (0.43-0.94) [0.0236] | 0.73 (0.48-1.13) [0.1554] | 0.8 (0.58-1.1) [0.1684] |
| 45_cpx | 36 | 0.88 (0.79-0.99) [0.0343] | 0.96 (0.85-1.09) [0.5415] | 0.91 (0.83-1) [0.0439] |
| 46_BF | 64 | 1 (0.92-1.09) [0.9683] | 0.97 (0.88-1.06) [0.4659] | 1.09 (1.02-1.17) [0.0148] |
| 47_BF | 7 | 0.75 (0.58-0.97) [0.0308] | 0.78 (0.59-1.04) [0.089] | 0.84 (0.68-1.03) [0.0941] |
| 49_cpx | 11 | 0.98 (0.8-1.2) [0.8456] | 1.25 (1-1.57) [0.0498] | 0.98 (0.82-1.15) [0.772] |
| A1 | 1138 | 0.77 (0.75-0.79) [<0.0001] | 0.97 (0.95-1) [0.0296] | 0.87 (0.85-0.89) [<0.0001] |
| A2 | 12 | 0.99 (0.81-1.21) [0.9159] | 0.94 (0.75-1.16) [0.5416] | 0.89 (0.76-1.05) [0.1724] |
| C | 7700 | 0.9 (0.89-0.92) [<0.0001] | 1.02 (1-1.04) [0.0228] | 0.91 (0.9-0.92) [<0.0001] |
| D | 784 | 0.81 (0.78-0.83) [<0.0001] | 0.91 (0.88-0.94) [<0.0001] | 0.86 (0.84-0.88) [<0.0001] |
| F1 | 381 | 0.8 (0.77-0.83) [<0.0001] | 0.83 (0.8-0.87) [<0.0001] | 0.86 (0.84-0.89) [<0.0001] |
| F2 | 48 | 0.71 (0.64-0.78) [<0.0001] | 0.79 (0.71-0.88) [<0.0001] | 0.83 (0.77-0.9) [<0.0001] |
| G | 147 | 0.91 (0.85-0.96) [0.0007] | 0.97 (0.92-1.04) [0.4158] | 0.97 (0.92-1.01) [0.1604] |
| H | 25 | 0.75 (0.65-0.86) [<0.0001] | 0.78 (0.67-0.91) [0.0013] | 0.83 (0.74-0.92) [0.0008] |
| J | 8 | 0.81 (0.64-1.04) [0.0932] | 0.91 (0.7-1.18) [0.4697] | 1.08 (0.89-1.32) [0.4246] |
| K | 8 | 0.72 (0.57-0.92) [0.0089] | 0.82 (0.63-1.07) [0.136] | 0.86 (0.7-1.04) [0.1205] |
| N | 8 | 0.68 (0.53-0.86) [0.0016] | 0.7 (0.54-0.91) [0.0075] | 0.85 (0.7-1.03) [0.1012] |
| O | 40 | 1.66 (1.49-1.85) [<0.0001] | 1.86 (1.66-2.1) [<0.0001] | 1.83 (1.67-2) [<0.0001] |
| P | 3 | 2.18 (1.46-3.23) [0.0001] | 2.15 (1.4-3.31) [0.0005] | 2.42 (1.76-3.34) [<0.0001] |
